# Supplementary material for: Phosphineoxide-Chelated Europium(III) Nanoparticles for Ceftriaxone Detection
Source: Nanomaterials (Basel). 2023 Jan 21;13(3):438. doi: 10.3390/nano13030438 (PMC9920168; doi:10.3390/nano13030438)
Supplement: Supplementary file 1 [file nanomaterials-13-00438-s001.zip › nanomaterials-2111291-supplementary.pdf]

Electronic Supplementary material

Title: Phosphineoxide-Chelated Europium(III) Nanoparticles for Ceftriaxone Detection

Authors: Rustem Zairov, Alexey Dovzhenko, Natalia Terekhova, Kornev Timur, Ying Zhou, Zeai Huang, Dmitry Tatarinov, Guliya Nizameeva, Robert R. Fayzullin, Aidar T. Gubaidullin, Taliya Salikhova, Francesco Enrichi, Vladimir F. Mironov, Asiya Mustafina

Content *Page*

*Synthesis and characterization of ligands **a**-**d*** 2

*General method for synthesis of complexes Ln(**a**) and Ln(**b**)* 3

Background for the analysis of the luminescence 4

Detailed crystallographic data 5

***Figure S1.** TEM image of PSS-[Eu(**b**)$_2$(NO$_3$)$_3$] and PSS-[Eu(**c**)(NO$_3$)$_3$(H$_2$O)]* 7

***Figure S2.** Gradual quenching of Eu^{3+} luminescence within the composition of PSS-[Eu(**a**)$_3$] colloids in the presence of increasing amounts of ceftriaxone (C=0,108 mM)* 8

References 9

*Synthesis and characterization of ligands **a-d***

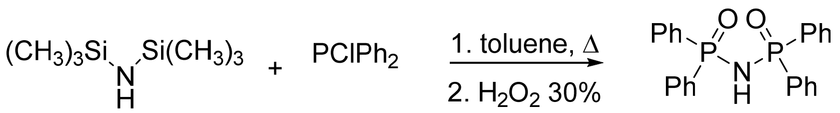

N-(diphenylphosphoryl)-*P*,*P*-diphenylphosphinic amide was obtained as described by S.W. Magennis [1]. A mixture of 1.1 g (10 mmol) Ph_2PCl and 0.8 g (5 mol) of hexamethyldisilazane in toluene (15 mL) was refluxed for 3 h. Then Me_3SiCl was removed by distillation. The reaction mixture was cooled by an ice bath and a solution of H_2O_2 (1 mL, aq 30 %) in THF (2 mL) was added dropwise. The resulting mixture was added to Et_2O (25 mL) giving white precipitate. This solid was washed several times with water and recrystallized from methanol to give *N*-(diphenylphosphoryl)-*P*,*P*-diphenylphosphinic amide (ligand **a**) as a white solid, yield 0.37 g (20 %). M.p. = 261-264°C; IR (KBr) ν = 3073, 3055, 1590, 1579, 1485, 1438, 1311, 1124, 1109, 1029, 763, 723, 696, 547, 527, 453, 432 cm^{-1}; ^{1}H NMR (400 MHz, $CDCl_3$) δ 7.80 – 7.73 (m, 8H), 7.49 (tq, $^{3}J_{HH}$ = 7.0, 1.6 Hz, 4H), 7.37 (td, $^{3}J_{HH}$ = 7.6, $^{4}J_{PH}$ = 3.7 Hz, 8H) ppm; ^{31}P NMR (162 MHz, $CDCl_3$) δ 25.7 ppm; ESI-MS m/z: = 418.12 $(M+H^{+})^{+}$

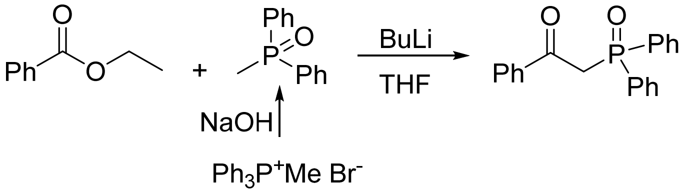

Methyldiphenylphosphine oxide was synthesized by procedure suggested by M.A. Gonzalez et al [2]. 2.5 g of methyltriphenylphosphonium bromide were dissolved in 25 mL of water and refluxed for 30 minutes. Then solution of 1.4 g of NaOH in 12.5 mL of water were added and stirring at reflux continued for 2 hours. After cooling solution was extracted with $CHCl_3$, organic layer washed with water, then dried over Na_2SO_4 and concentrated. Slightly yellow solid. Off-white solid after recrystallization from Et_2O, yield 1.11 g (77.4%). ^{1}H NMR (400 MHz, $CDCl_3$) δ 7.78 – 7.66 (m, 4H), 7.54 – 7.48 (m, 2H), 7.48 – 7.42 (m, 4H),, 2.01 (d, $^{2}J_{PH}$ = 13.2 Hz, 3H) ppp; ^{31}P NMR (162 MHz, $CDCl_3$) δ 30.5 ppm;

According to procedure suggested by J.S. Maass et al. [3]. 0.7 g of methyldiphenylphosphine oxide were dissolved in 30 mL of THF and 1.27 mL of 1M butyl lithium in hexane were added at 0°C. The reaction mixture was cooled to – 78°C and ethyl benzoate added dropwise. After that the reaction mixture allowed to warm to r.t. Upon reaction considered complete in 3 hours it was quenched with saturated ammonium chloride solution, the solvent evaporated.2-(diphenylphosphoryl)-1-phenylethan-1-one (ligand **b**), white solid, yield 0.42 g (40.5%) M.p. = 138-140 °C; IR (KBr) ν = 3053, 2876, 1681 (C=O), 1595, 1580, 1440 (CH2), 1373, 1323, 1296, 1181 (P=O), 1146, 1118, 1101, 1075, 990 cm^{-1}; ^{1}H NMR (600 MHz, $CDCl_3$) δ 7.97 (d, $^{3}J_{HH}$ = 7.7 Hz, 2H), 7.79 (dd, J = 12.2, $^{3}J_{HH}$ = 7.5 Hz, 4H), 7.56 – 7.33 (m, 9H), 4.13 (d, 2H, $^{2}J_{PH}$ = 15.3 Hz, CH_2) ppm; ^{31}P NMR (243 MHz, $CDCl_3$) δ 26.9 ppm.

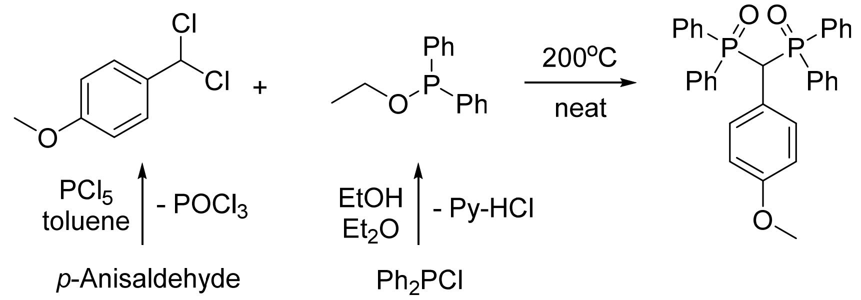

Ligand **c** was obtained by reaction of 0.43 mL (0.46 g, 2 mmol) of ethyl diphenylphosphinite and 0.17 g (0.9 mmol) of 95% 1-(dichloromethyl)-4-methoxybenzene and heated neat till 200°C at which point the reaction mixture solidifies. The solid obtained was recrystallized from CH_3CN, and washed with Et_2O. ((4-methoxyphenyl)methylene)bis(diphenylphosphine oxide) obtained as a white solid, yield 0.16 g (16.4 %) M.p. = 313-315°C; IR (KBr) ν = 3073, 3055, 1591, 1579, 1485, 1438, 1336, 1311, 1183, 1124, 1109, 1029, 763, 723, 694, 547, 527, 452, 432 cm^{-1}; ^{1}H NMR (400 MHz, $CDCl_3$) δ 7.79 – 7.69 (m, 4H), 7.56 – 7.48 (m, 4H), 7.33 – 7.28 (m, 4H), 7.25 – 7.18 (m, 6H), 7.12 (td, *J* = 7.6, 3.3 Hz, 4H), 6.43 (d, *J* = 8.9 Hz, 2H), 4.75 (t, *J* = 15.4 Hz, 1H), 3.56 (s, 3H) ppm; ^{31}P NMR (162 MHz, $CDCl_3$) δ 29.0 ppm; ESI-MS m/z: = 523.18 $(M+H^{+})^{+}$ and 545.15 $(M+Na^{+})^{+}$

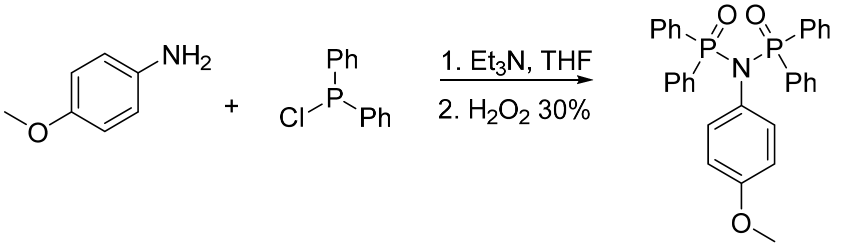

Bis(diphenylphosphanyl)(4-methoxyphenyl)amine was synthesized according to T. Ogawa et al [4]. 246 mg of 4-methoxyaniline were dissolved in THF (5 mL) and in presence of 2 eq. of Et_3N (0.56 mL) chlorodiphenylphosphine mixture in THF (5 mL) was added via dropping funnel at 0°C and left stirring overnight. $Et_3N{\cdot}HCl$ was filtered off and the residue was concentrated *in vacuo* and used directly in the next step. 600 mg of the crude product were dissolved in acetone and cooled to 0°C. Then 1 mL of H_2O_2 (aq 30%) were added dropwise and let stirring for an hour. After that reaction mixture was concentrated *in vacuo* and recrystallized in the freezer from methanol giving *N*-(diphenylphosphoryl)-*N*-(4-methoxyphenyl)-*P*,*P*-diphenylphosphinic amide as a brownish solid. Second recrystallization from a few droplets of dichloromethane and hexane gave Ligand **d** as a white solid. Yield 0.14 (21.9%). M.p. = 217-219°C; IR (KBr) ν = 3077, 3055, 1439, 1192, 1180, 1128, 958, 756, 728, 694, 551, 525, 500, 436 cm^{-1}; ^{1}H NMR (400 MHz, $CDCl_3$) δ 7.93 – 7.75 (m, 8H), 7.30 – 7.22 (m, 8H), 7.22 – 7.13 (m, 6H), 6.43 (d, $^{3}J_{HH}$ = 8.9 Hz, 2H), 3.55 (s, 3H) ppm; ^{31}P NMR (162 MHz, $CDCl_3$) δ 24.3 ppm; ESI-MS m/z: = 524.20 $(M+H^{+})^{+}$ and $(Ph_2POH)^{\cdot+}$ = 202.93;

General method for synthesis of complexes Ln(a) and Ln(b)

As an example, given procedure for Ligand **a**. 26.1 mg (0.05 mmol) of Ligand **a** were dried *in vacuo* to ensure absence of water. The ligand dissolved in dry THF added and equivalent amount of NaH disperse in mineral oil (2 mg) added. After reaction considered complete (gas evolution

stops), reaction mixture was concentrated and dried *in vacuo*. The resulting mixture was dissolved in dry CH_3CN and mixed with separately dried 7,1 mg of $Ln(NO_3)_3$ while stirring.

Background for the analysis of the luminescence

In 1962, Judd [5] and Ofelt [6] independently developed a model for the calculation of the oscillator strengths of lanthanide electric dipole f-f transitions. Judd–Ofelt theory has been very successful in understanding and predicting the spectral intensities, especially for ions in inorganic glasses and crystals.

The red luminescence of Eu^{3+} is a result of transitions from its $^5D_0$ state to the lower lying ^7F_J levels (J = 0-6). The transitions from $^5D_0$ to $^7F_{0,3,5}$ are forbidden both in magnetic and induced electric dipole schemes. The transition to $^7F_1$ is the only magnetic dipole contribution. Magnetic dipole transitions in lanthanide ions are practically independent of the ion's surroundings and can be well calculated by theory. The remaining transitions to $^7F_{2,4,6}$ are purely of induced electric dipole nature. According to the Judd–Ofelt theory, the strength of all induced dipole transitions (absorption and emission) of a lanthanide ion in a certain matrix can be calculated on basis of only three parameters, which are usually determined experimentally.

Transitions arising from the $^5D_0$ level of the ^4f_J electronic configuration of Eu^{3+} are easily assigned by the theory. The first important observation concerns the relative transition intensities between the electric dipolar $^5D_0 \rightarrow {}^7F_2$ (about 610 nm) and the magnetic dipolar $^5D_0 \rightarrow {}^7F_1$ (about 590 nm) transitions:

$$R = \frac{I(\,^5D_0 \rightarrow \,^7F_2)}{I(\,^5D_0 \rightarrow \,^7F_1)} \qquad (1)$$

The higher this ratio, the lower the local symmetry around Eu^{3+} is with respect to an inversion centre, since a high local symmetry strongly reduces the electric dipolar emission without affecting the magnetic dipolar one, and vice versa. In particular, Reisfeld et al. reported several studies on different europium environments, such as glasses, crystals, complexes and solutions, which are summarized in [7], demonstrating that this indicator can be lower than 1 when the ion is situated in a totally symmetric environment such as water or cubic crystals, and it increases above 1 in solidified glasses, where the modifier ions are responsible for the distortion of the oxygens around the RE ions lowering the site symmetry and increasing the probability for f–f transitions. Also, in many complexes the ratio is high due to increase of covalency of Eu with the surrounding ligands, which lowers the symmetry.

Another unique property related to the internal ^{4}f transitions of Eu^{3+} ions as depicted by the Judd-Ofelt analysis is that the intensity of the $^5D_0 \rightarrow {}^7F_1$ transition, being magnetic dipolar in character and therefore insensitive to change in the Eu^{3+} surroundings, may be taken as a reference, with a radiative rate of 50 s^{-1}. All the other observed transitions arising from the $^5D_0$ level have electric dipolar character. Therefore, assuming the occurrence of only radiative and non-radiative processes in the depopulation of the $^5D_0$ excited level, from these two intensity ratios the $^5D_0$ radiative lifetime can be evaluated. From a practical point of view, the radiative lifetime (τ_{rad}) can be calculated by the following equation:

$$\left(\frac{1}{\tau_{rad}}\right)_{Eu} = 14.65\, n^3 \frac{I(\,^5D_0 \rightarrow \,^7F_J)}{I(\,^5D_0 \rightarrow \,^7F_1)}, \qquad (2)$$

where n indicates the refractive index, $I(^5D_0 \rightarrow {}^7F_J) / I(^5D_0 \rightarrow {}^7F_1)$ is the ratio between the total integrated emission from the Eu $^5D_0$ level to the ^7F_J manifold (J = 0–6) and the integrated intensity of the transition $^5D_0 \rightarrow {}^7F_1$ [8,9].

Therefore, by comparing the experimentally measured lifetime value τ_{meas} with the radiative lifetime calculated from the luminescence emission spectra τ_{calc} it is possible to give an evaluation of the quantum efficiency of the emitting Eu^{3+} ions by means of the following equation:

$$\phi = \frac{\tau_{meas}}{\tau_{calc}} \qquad (3)$$

Detailed crystallographic data

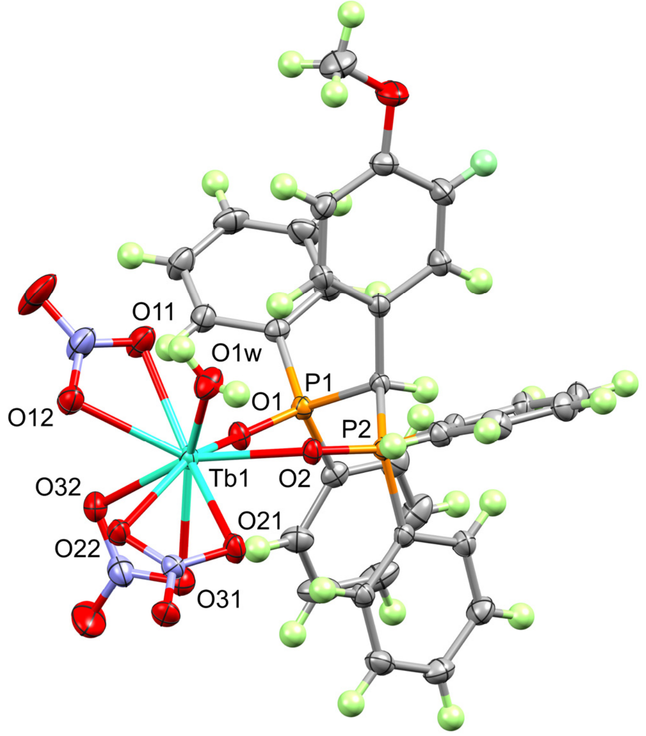

ORTEP of complex Tb(**c**) at the 50 % probability level for non-hydrogen atoms according to single-crystal X-ray diffraction. THF solvent molecules are omitted for clarity. Selected internuclear distances [Å]: Tb1–O1 2.3680(14), Tb1–O2 2.2949(13), Tb1–O1w 2.3532(18), Tb1–O11 2.4298(16), Tb1–O12 2.5068(15), Tb1–O21 2.4976(15), Tb1–O22 2.4872(15), Tb1–O31 2.4653(16), Tb1–O32 2.4351(15), P1–O1 1.5047(14), P2–O2 1.5001(14).

Single crystals of complex Tb(**c**) as colorless prisms were prepared by slow evaporation of a THF solution. The compound crystallizes in the monoclinic space group *C*2/*c* as a crystallosolvate with 2.5 THF molecules per complex. One complex molecule is present in the asymmetric cell. Its molecular structure is shown in Figure 1 and reflects the 1:1 stoichiometry. Nine-coordinated Tb atom is surrounded by one ligand **c** attached by bisphosphine oxide chelation

through the P=O coordination, three ditopic NO_3^- residues, and one water molecule. Interestingly, the [P=]O–Tb internuclear distances are notably different and equal to 2.3680(14) and 2.2949(13) Å. The internuclear distances between the Eu and coordinated oxygen atoms of NO_3^- vary between 2.4298(16) and 2.5068(15) Å.

X-ray structures of previously studied Tb(**a**)$_3$ and Eu(**b**)$_2$ and newly obtained Tb(**c**) are illustrated in Figure 2. Complex Tb(**a**)$_3$ crystallizes with two molecules in the asymmetric cell. Although both molecules are situated at the crystallographic 3-fold proper rotating axes, they show different coordination modes, namely, trigonal prismatic and octahedral. Complexes Eu(**b**)$_2$ and Tb(**c**) are located in general position in the crystals and, consequently, they are asymmetric.

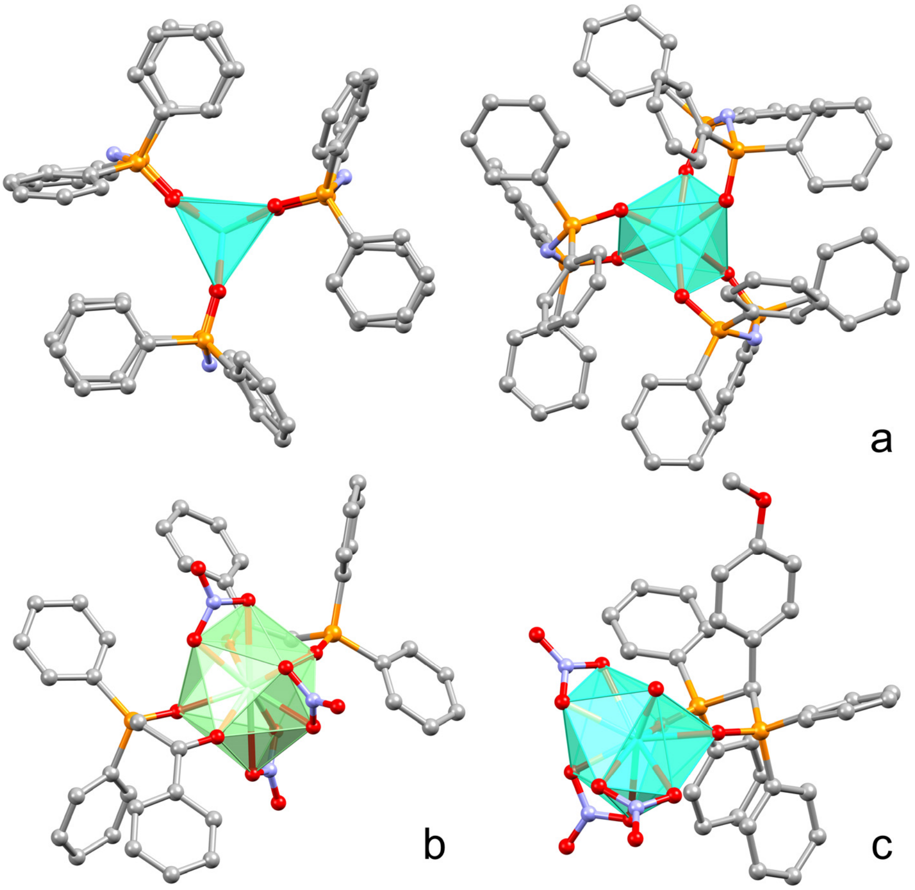

Comparison of the X-ray structures of complexes Tb(**a**)$_3$ (a), Eu(**b**)$_2$ (b), and Tb(**c**) (c). Metal atoms are shown as polyhedra. Hydrogen atoms are omitted for clarity. In the case of Tb(**a**)$_3$, both molecules represented in the asymmetric cell are shown.

X-ray diffraction study of Tb(**c**) was performed on a Bruker D8 QUEST diffractometer. Data collected were processed using the *APEX*4 software. The structure was solved by the direct method using the *SHELXT* program[10] and refined by the full-matrix least-squares method on F^2 using the *SHELXL* program[11]. Non-hydrogen atoms were refined in the anisotropic

approximation. The positions of hydrogen atoms H11w and H12w of the coordinated water molecule were determined using difference Fourier maps, and these atoms were refined isotropically. The other hydrogen atoms were inserted at geometrically calculated positions and included in the refinement as riding atoms.

Deposition number CCDC 2218909 contains the supplementary crystallographic data for compound Tb(**c**). These data are provided free of charge by the joint Cambridge Crystallographic Data Centre and Fachinformationszentrum Karlsruhe Access Structures service www.ccdc.cam.ac.uk/structures.

Crystallographic data for Tb(**c**). $C_{42}H_{50}N_3O_{15.5}P_2Tb$, colorless prism (0.341 × 0.150 × 0.146 mm^3), formula weight 1065.71 g mol^{-1}; monoclinic, *C*2/*c* (No. 15), a = 43.8397(18) Å, b = 10.6969(5) Å, c = 20.2296(9) Å, β = 110.7701(9)°, V = 8870.1(7) Å^3, Z = 8, Z' = 1, T = 150(2) K, d_{calc} = 1.596 g cm^{-3}, μ(Mo Kα) = 1.740 mm^{-1}, F(000) = 4336; $T_{max/min}$ = 0.7616/0.5180; 195864 reflections were collected (1.968° ≤ θ ≤ 29.574°, index ranges: –60 ≤ h ≤ 60, –14 ≤ k ≤ 14 and –28 ≤ l ≤ 28), 12432 of which were unique, R_{int} = 0.0628, $R_σ$ = 0.0270; completeness to θ of 29.574° 100.0 %. The refinement of 641 parameters with 317 restraints converged to $R1$ = 0.0258 and $wR2$ = 0.0555 for 9412 reflections with $I > 2σ(I)$ and $R1$ = 0.0435 and $wR2$ = 0.0610 for all data with goodness-of-fit S = 1.042 and residual electron density $ρ_{max/min}$ = 0.441 and –0.617 e Å^{-3}, rms 0.085; max shift/e.s.d. in the last cycle 0.004.

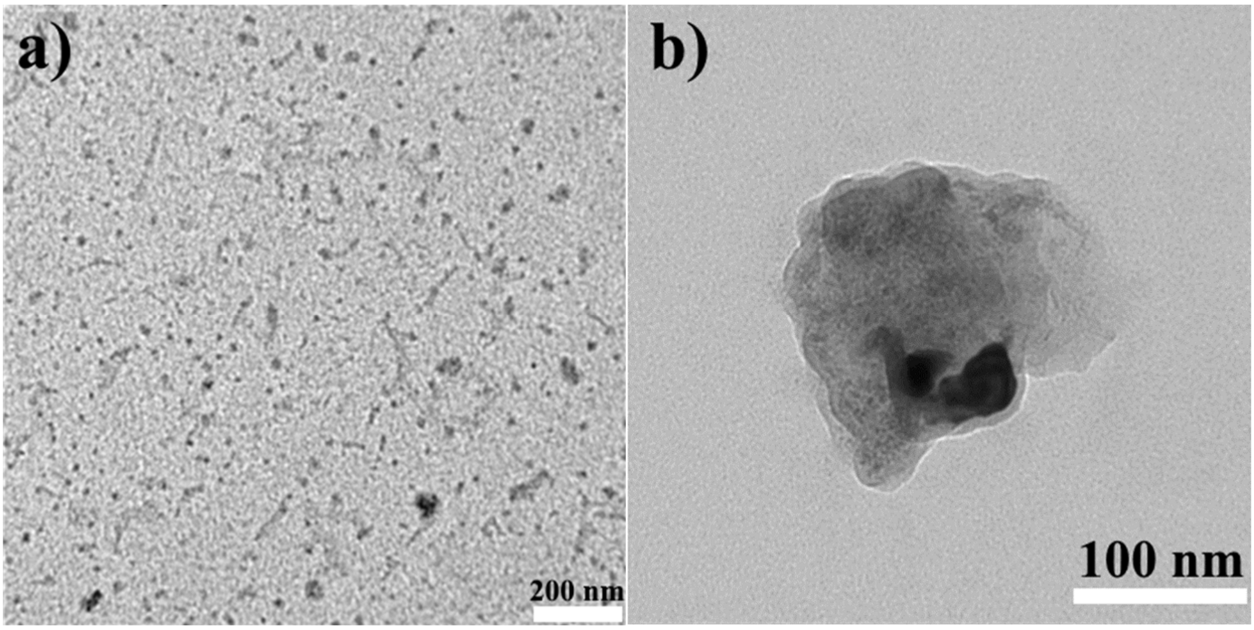

Figure S1. TEM image of PSS-[Eu(**b**)$_2$(NO$_3$)$_3$] (a) and PSS-[Eu(**c**)(NO$_3$)$_3$(H$_2$O)] (b).

.

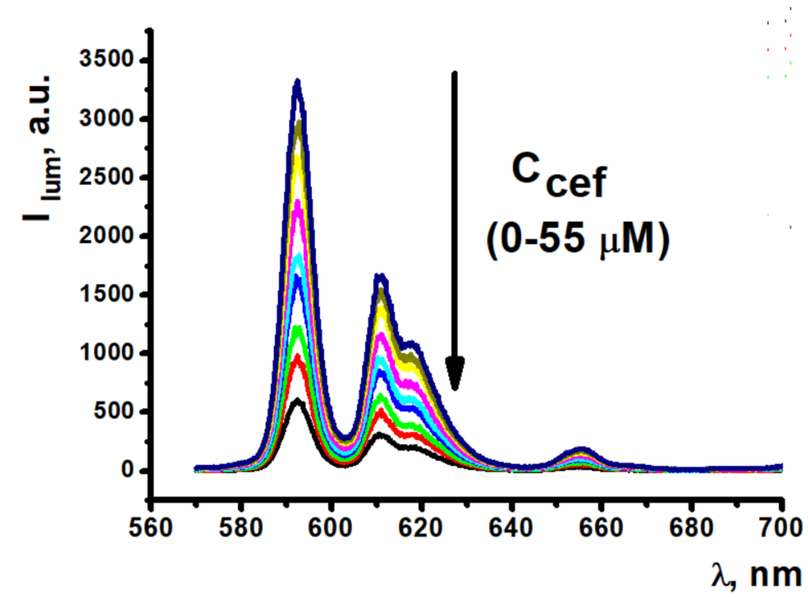

Figure S2. Gradual quenching of Eu^{3+} luminescence within the composition of PSS-[Eu(**a**)$_3$] colloids in the presence of increasing amounts (0-3 equivalents) of ceftriaxone (C=0,108 mM).

References

1. Magennis, S.W.; Parsons, S.; Pikramenou, Z. Assembly of Hydrophobic Shells and Shields around Lanthanides. *Chem. - A Eur. J.* **2002**, *8*, 5761–5771, doi:10.1002/1521-3765(20021216)8:24<5761::AID-CHEM5761>3.0.CO;2-H.

2. Gonzalez, M.A.; Walker, A.S.; Cao, K.J.; Lazzari-Dean, J.R.; Settineri, N.S.; Kong, E.J.; Kramer, R.H.; Miller, E. W. Voltage Imaging with a NIR-Absorbing Phosphine Oxide Rhodamine Voltage Reporter. *J. Am. Chem. Soc.*, **2021**, *143*, 2304–2314. doi:10.1021/jacs.0c11382

3. Maass, J.S.; Wilharm, R.K.; Luck, R.L.; Zeller, M. Photoluminescent properties of three lanthanide compounds of formulae LnCl3(diphenyl((5-phenyl-1H-pyrazol-3-yl)methyl)phosphine oxide)2, Ln = Sm, Eu and Tb: X-ray structural, emission and vibrational spectroscopies, DFT and thermogravimetric studies. *Inorganica Chim. Acta* **2018**, *471*, 481–492, doi:10.1016/j.ica.2017.11.049.

4. Ogawa, T.; Kajita, Y.; Wasada-Tsutsui, Y.; Wasada, H.; Masuda, H. Preparation, Characterization, and Reactivity of Dinitrogen Molybdenum Complexes with Bis(diphenylphosphino)amine Derivative Ligands that Form a Unique 4-Membered P–N–P Chelate Ring. *Inorg. Chem.* **2013**, *52*, 182–195, doi:10.1021/ic301577a.

5. Judd, B.R. Optical absorption intensities of rare-earth ions. *Phys. Rev.* **1962**, *127*, 750–761, doi:10.1103/PhysRev.127.750.

6. Ofelt, G.S. Intensities of crystal spectra of rare-earth ions. *J. Chem. Phys.* **1962**, *37*, 511–519, doi:10.1063/1.1701366.

7. Reisfeld, R.; Zigansky, E.; Gaft, M. Europium probe for estimation of site symmetry in glass films, glasses and crystals. *Mol. Phys.* **2004**, *102*, 1319–1330, doi:10.1080/00268970410001728609.

8. Malba, C.; Sudhakaran, U.P.; Borsacchi, S.; Geppi, M.; Enrichi, F.; Natile, M.M.; Armelao, L.; Finotto, T.; Marin, R.; Riello, P.; et al. Structural and photophysical properties of rare-earth complexes encapsulated into surface modified mesoporous silica nanoparticles. *Dalt. Trans.* **2014**, *43*, 16183–16196, doi:10.1039/c4dt00760c.

9. Werts, M.H. V.; Jukes, R.T.F.; Verhoeven, J.W. The emission spectrum and the radiative lifetime of Eu3+ in luminescent lanthanide complexes. *Phys. Chem. Chem. Phys.* **2002**, *4*, 1542–1548, doi:10.1039/b107770h.

10. Sheldrick, G.M. SHELXT – Integrated space-group and crystal-structure determination. *Acta Crystallogr. Sect. A Found. Adv.* **2015**, *71*, 3–8, doi:10.1107/S2053273314026370.

11. Sheldrick, G.M. Crystal structure refinement with SHELXL. *Acta Crystallogr. Sect. C Struct. Chem.* **2015**, *71*, 3–8, doi:10.1107/S2053229614024218.
